# Chronicle of a Pigmented Superficial Basal Cell Carcinoma

**DOI:** 10.1155/2012/693041

**Published:** 2012-07-08

**Authors:** Marie Caucanas, Claudine Piérard-Franchimont, Gérald E. Piérard

**Affiliations:** ^1^Department of Dermatology, Regional Hospital of Huy, Huy, Belgium; ^2^Department of Dermatopathology, University Hospital of Liège, 4000 Liège, Belgium; ^3^University of Liège, Liège, Belgium

## Abstract

Dermoscopic patterns of basal cell carcinoma (BCC) are well defined, but the dynamics of dermoscopic changes in time were apparently never described so far. In this paper, prominent changes were observed over a 8-week period, allowing to establish a close connection between spoke wheel areas and maple leaf-like aspects, through progressive thickening of the former ones. A chronobiological phenomenon ruling synchronous apoptosis in some of the most superficial BCC nests is suggested, leading to a wax and wane process of millimetric crusts, taking part in the spontaneous BCC regression/progression process.

## 1. Report

**Figure 1:**
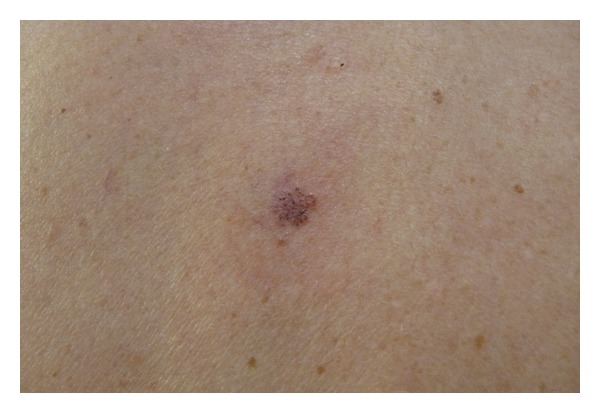
Crusty pigmented lesion located in the dorsal region.

**Figure 2:**
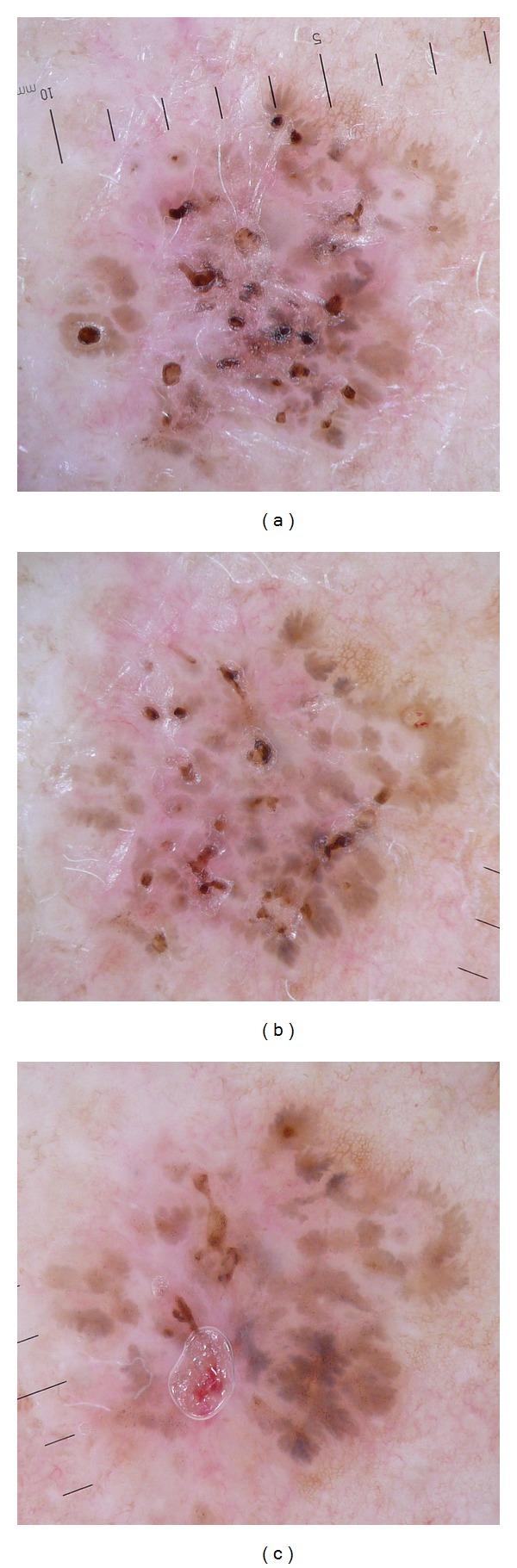
(a) Initial appearance under Heine 20 dermoscopy. (b) Dermoscopic examination 6 weeks later, before biopsy. (c) Dermoscopic examination 8 weeks later, after biopsy stitch removal.

A pigmented discretely crusty lesion of the back (Figure 1) was unexpectedly disclosed during clinical examination in a 69-year-old woman, who consulted for a Darier disease followup. The lesion reached about 8 mm in diameter. It was brownish without obvious inflammation. Examination with a Heine 20 dermatoscope (Herrsching, Germany) showed small superficial erosions centrally located in radial pigmented structures looking needle-shaped spoke wheels particularly present in the periphery of the lesion (Figure 2(a)). In addition, some aspects of maple leaf-like structures were found, particularly in the right lower part, altogether such dermoscopic clues evoked the diagnosis of a pigmented superficial basal cell carcinoma (BCC). A biopsy was planned six weeks later. 

A second dermoscopic examination performed before the skin sampling revealed the thickening of spoke wheel areas representing transitional forms towards maple leaf-like aspects. The numerous small crusts had vanished (Figure 2(b)).

The histopathological BCC diagnosis confirmed the clinical suggestion. After stitch removal two weeks later, a final dermoscopic examination was carried before performing complete BCC excision. A marked thickening of the pigmented structures was observed with well-structured maple leaf-like areas, particularly obvious in the right lower part (Figure 2(c)).

This dermoscopic observation showed an evident evolution of the morphology of a superficial BCC over a period of 8 weeks only. To the best of our knowledge, such dynamics had never been described so far.

The clinical [1], histopathological [2, 3], and pathobiological [4] evolving aspects of BCC were subject to some previous publications, as well as several observations allowing to establish a correlation between the dermoscopic structures and their microscopic counterparts [5–8].

Hirofuji et al. [5] recently described the progressive extension of spoke wheel areas leading to transitional “petal-like” forms or to maple leaf-like aspect, which is consistent with the present dermoscopic observation.

During the present dermoscopic followup, the small crusts seen at the beginning had all disappeared in a relatively short period of time. Some clinicians would attribute the erosions to the extreme friability of the BCC. This opinion is not supported by sound biological features. It is better assumed that millimetric crusts were likely the issue of an underlying mechanism of apoptosis and necrosis involving small tumoral nests appended to the epidermis. Both the synchronous arisen and recovery of the crusting process evoke a chronobiological phenomenon affecting a set of focal tumoral spots. An environmental effect, such as a microtrauma by friction or desiccation of the thinned skin covering the BCC nests, cannot be ruled out.

Occasional signs of progression and regression of BCC have already been described [9]. Their balance explains why superficial BCC has a global slow growth rate despite a high number of cells engaged in the cell cycle of proliferation. This phenomenon of small-scale spontaneous regression could be put in parallel with the regression induced by topical applications of imiquimod, whose mechanisms of action imply autophagy as well as apoptosis [10]. Their intensity is sometimes such that crusty or even necrotic lesions tend to occur.

In short we presently report the dynamic dermoscopic patterns of a BCC in a matter of 8 weeks. This first documented photographic evolution of dermoscopic features calls for larger studies exploring the apparently unique way of tumoral BCC progression and therapeutic regression.
